# Barriers for early initiation and exclusive breastfeeding up to six months in predominantly rural Sri Lanka: a need to strengthen policy implementation

**DOI:** 10.1186/s13006-021-00378-0

**Published:** 2021-04-08

**Authors:** Thilini Chanchala Agampodi, Neerodha Kithmini Dharmasoma, Iresha Sandamali Koralagedara, Thushari Dissanayaka, Janith Warnasekara, Suneth Buddhika Agampodi, Rafael Perez-Escamilla

**Affiliations:** 1 Department of Community Medicine, Faculty of Medicine and Allied Sciences, Rajarata University of Sri Lanka, Anuradhapura, Sri Lanka; 2 Department of Social and Behavioral Science, Yale School of Public Health, New Haven, USA

**Keywords:** Exclusive breastfeeding, Early breastfeeding initiation, Sri Lanka, Qualitative

## Abstract

**Background:**

Sri Lanka was named as the first-ever ‘Green’ breastfeeding nation status by the World Breastfeeding Trends Initiative (WBTi) in January 2020. However, improvements are still needed. This study aims to identify barriers and facilitators for early initiation of breastfeeding and exclusive breastfeeding for 6 months in rural Sri Lanka.

**Methods:**

We conducted in-depth interviews with 16 mothers with infants, who had been unable to practice early initiation of breastfeeding and/or exclusive breastfeeding (EBF), in six child-welfare clinics in Anuradhapura, Sri Lanka. Three focus group discussions were held with public health midwives (PHMs). Initial thematic analysis that built upon force field and social learning theories was performed.

**Results:**

Main barriers for EBF were clustered at three time periods; during the first 2–3 days, 2–3 weeks, and 4–5 months postpartum. Early barriers included cesarean section pain, poor breast latch, maternal exhaustion, suboptimal maternity ward environment, and lack of support for breastfeeding. Around 2–3 weeks postpartum mothers introduced water or infant formula due to social norms and poor support. On-demand feeding was misunderstood. Around 4 and 5 months postpartum, EBF ended due to return to work. PHMs reported a heavy workload limiting their time to support breastfeeding.

**Conclusion:**

EBF interruption was due to diverse individual- and environnmental- level barriers that varied across the first 6 months. To improve EBF, Sri Lanka should focus on strengthening policies for reducing the excessive rates of cesarean section, improving support in maternity ward facilities, fostering on-demand breastfeeding, enhancing support for working mothers and reducing the work load of PHMs.

**Supplementary Information:**

The online version contains supplementary material available at 10.1186/s13006-021-00378-0.

## Background

Breastfeeding is the single most cost-effective intervention for reducing morbidity and mortality in children [1]. The global recommendations for breastfeeding include initiation of breastfeeding during the first hour after birth, practicing exclusive breastfeeding (EBF) up to 6 months, continuing breastfeeding for at least 2 years and practicing on-demand feeding [2]. Approximately 7.7 and 19.1% of all neonatal deaths may be avoided by the universal initiation of breastfeeding within the first day or first hour of life, respectively [3]. Breastfeeding is key for mother–child bonding and promotes sensory and cognitive development [2, 4]. Skin to skin contact since birth provides a nurturing environment that allows for early breastfeeding initiation, provides protection from hypothermia, and promotes the establishment of a healthy microbiota in the newborn [2]. Current scientific evidence indicates that breastfeeding is associated with lower rates of infections among infants [5] including diarrhea [6], respiratory tract infections [6], otitis media [7] and allergic rhinitis [8]. Furthermore, breastfeeding has been associated with higher levels of intelligence [9], and reduced risk of future onset of diabetes mellitus and obesity [10]. As a result, breastfeeding provides strong economic benefits to the family and society at large [11]. In 2016, the United Nations reaffirmed EBF as a human right and called for all countries to keep close oversight of marketing practices of infant formula companies [12].

Sri Lanka is considered to be a global role model for advancing breastfeeding protection, promotion and support. This is reflected in the fact that the country is the first ever to have received a ‘Green status’ by the World Breastfeeding Trends Initiative (WBTi) [13]. This outcome has been achieved in the context of strong political commitment in a country with a Gross National Income (GNI) per capita of USD$4020 [14] and health expenditures of less than 5% of Gross Domestic Product (GDP). The main pillars of maternal-child health are connected to free education, free and efficient clinical and public healthcare services, and a strong emphasis on gender equity.

The origin of the current public health legislation and services in Sri Lanka dates back to the late eighteen hundreds, with the health unit system for maternal health services initiated in 1926 [15]. The 362 Medical Officer of Health (MOH) areas distributed all over the country are the main public health administrative units; with each area serving a population of about 60,000 individuals. The individual in charge of these areas are medical doctors with training on public health. Each MOH oversees public health staff including supervisory officers and grass root level primary healthcare officers. The grass root level maternal and child health workers in this system are public health midwives (PHMs). The PHMs register eligible families, offer preconceptual, prenatal and postpartum care in in field clinics and at home. PHMs are a key part of the strong Sri Lankan government network tasked with promoting, protecting and supporting breastfeeding.

In response to the widespread introduction and marketing of infant formula in the 1960–70s, the Sri Lankan government began taking actions to protect breastfeeding in 1979, and in 1980 the advertising of infant formula via mass media was banned. The WHO Code for Marketing of Breastmilk Substitutes was endorsed by Sr Lanka and the Sri Lankan Code for Promotion of Breast Feeding and Marketing Breast Milk Substitutes and Products [16] was issued in 1981 with subsequent revisions in 1998 and 2002. In 1993, the Baby Friendly Hospital Initiative (BFHI) was adopted in Sri Lanka. The Lactation Management Centers in hospitals were established in 2005 to promote and support breastfeeding counseling and rooming-in [17]. The Family Health Bureau under the Ministry of Health implemented National in-service breastfeeding training programs for public health and hospital staff involved in breastfeeding support. Women working in the government service are covered by paid maternity leave for 84 working days after delivery, a half paid leave for another 84 days, with the option of additional 84 days of non-paid leave.

The latest Demographic and Health Survey (DHS) conducted in 2016 indicates that 99% of the children are ever breast fed in Sri Lanka. The prevalence of early initiation of breastfeeding is 90% and the percentage of newborns offered the breast within 1 h after birth range from 77% in Mannar to 100% in Anuradhapura. The prevalence of EBF declines from 93% at 0–1 months, to 87% at 2–3 months and to 64% at 4–5 months [18].

However, there are still important actions that need to take place for breastfeeding improvements to continue. The purpose of this qualitative study is to identify individual, and environmental (i.e., healthcare and social systems) barriers that prevent more women from practicing early initiation of breastfeeding and exclusive breastfeeding. Findings from this study are likely to help inform the development or strengthening of more inclusive breastfeeding policies and programs in Sri Lanka.

## Methods

### Study design

This qualitative study included in-depth interviews with mothers with infants who did not practice early initiation of breastfeeding or EBF up to six months, and also used focus group discussions with public health midwives (PHMs) in a predominantly rural area of Sri Lanka. This study was conducted from September 2017 to January 2018.

### Study setting

The study was conducted in Anuradhapura, the largest district in Sri Lanka (7179 sqm). The total population in the district is 886,945 of which 94.1% is rural [19]. In this district more than 17,000 pregnant mothers are registered annually for antenatal care [20] and DHS data shows that antenatal care coverage through the public health system is 100%. Almost all deliveries (98%) take place in health institutions [18] and 90% of females in the district have at least entered secondary level education. This study was conducted in three selected MOH areas out of a total of twenty-two areas. The selection was purposeful, based on the different types of barriers anticipated in different communities. The three MOH areas were Nuwaragampalaatha Central (NPC) (rural), Nuwaragampalaatha East (NPE) (Urban) and Rajanganaya (resettled).

### Participants

Study participants included mothers of infants with a history of interruption of early initiation of breastfeeding or EBF before six months. PHMs of the three areas were selected to participate in focus groups discussions (FGDs) to explore their experience with mothers who were under their care and were not able to do early initiation and exclusive breastfeeding. The FGDs also focused on understanding maternity facility and social barriers for early initiation of breastfeeding and exclusive breastfeeding.

### Sampling and recruitment of participants

Six polyclinics were selected from the three MOH areas, based on data collection feasibility considerations. Of the mothers attending the routine polyclinic services on the day of data collection, all those with delayed breastfeeding initiation or interruptions of EBF before completion of six months were selected, with the aid of the PHM and a short screening form (see Appendix 1), and invited to participate in the study. Informed written consent was obtained from the mothers. Caretakers other than the mother were excluded. Every MOH conducted a monthly conference and an in-service training, where all PHMs and other public health officers in the whole MOH area gathered at the MOH office. At the monthly conferences the servicesprovided by all public health staff from the MOH area are reviewed. At hese meetings updates on current and new public health services delivered by the Ministry of health are discussed. In-service training day is a compulsory monthly meeting for all public health staff in the MOH where they are offered training on selected topics to support continuous professional development. PHMs from the three MOH areas, were invited  to participate in the FGDs that took place in a convenient room in the MOH office at a free time during the monthly conference or in-service training day. Full information about the study was given to all study participants before informed verbal consent was obtained.

### Data collection

#### Data collection instruments

Interviewer guides for in-depth interviews with mothers and for FGDs with PHMs (see Appendices 2, 3 and 4) were developed using standard protocols and guidelines [21]. Probing questions were developed to explore the areas of interest regarding barriers of early initiation of breastfeeding and EBF.

#### Training of data collectors

Data collectors were pre-intern medical officers of the Department of Community Medicine in the Faculty of Medicine and Allied Sciences, Rajarata University of Sri Lanka. They were trained for qualitative interview techniques, data collection and safe storage according to standard guidelines [22].

#### Data collection procedure

After obtaining informed written consent, data collectors conducted in depth interviews with mothers selected in the routine immunization clinic or polyclinic, at a convenient time for the participants. The interviews were conducted before each child vaccination in a private location in the clinic. Each interview lasted twenty to thirty minutes. FGDs were conducted at a convenient room in the MOH office. A moderator conducted the interviews while a note taker took down notes. Every PHM in the group was given the chance to describe her own experience on the topics discussed. The interviews and FGDs were tape recorded. At the end of the sessions, a summary of the recorded data was presented to participants for respondent validation. The summary provided was based on the notes taken down during the interviews and focus group discussions. The interview/FGD notes were expanded immediately after the interviews. The recorded data was transcribed within the first few days of the interview/focus group discussions. Transcription, organization and storage of data were done according to recommendations [22].

### Data analysis

Three investigators conducted the analysis. Two investigators independently coded data based on a coding scheme used to code all interview transcripts that was developed after consensus was reached by all three investigators. Barriers to early initiation of breastfeeding and EBF were identified from the interviews with the mothers as well as from the PHMs in the focus group discussions. Themes were identified inductively initially and then they were categorized using driving and restraining forces according to Force Field theory [23]. Next, the themes that emerged were further collated and categorized into major themes according to social learning theory [24] to identify individual- and environmental- level factors.

### Quality assurance

We used standard techniques and guides for all data collection procedures. Respondent validation was used at the end of each interview. Triangulation was performed using in-depth interviews of mothers and FGDs of PHMs. Reflexivity was addressed by maintaining a diary by the investigators. Independent coding was performed, and the codes and themes were identified following a consensus approach.

## Results

**Fig. 1:**
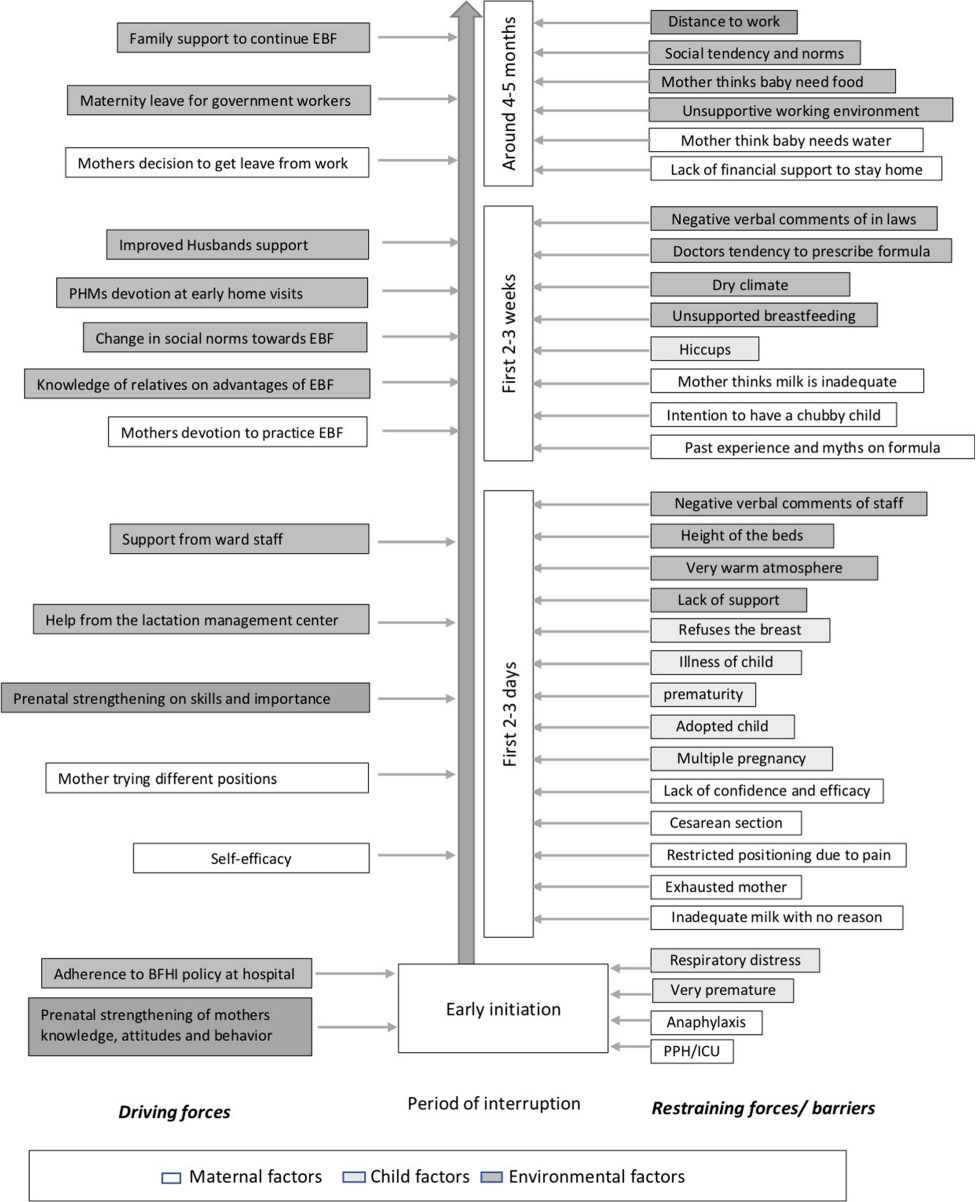
Force field analysis of barriers and facilitators for early initiation of breastfeeding and EBF at critical time periods relating to personal and environmental factors

Sixteen mother-infant pairs (eight mothers with less than six months old children and ten mothers with infants between six months to one year of age) and 34 PHMs participated in the study. Mothers’ ages ranged from 21 to 40 years. All mothers except one were educated up to or beyond grade eleven. Four mothers were working and the remaining were housewives. The force field analysis of barriers and facilitators for early initiation of breastfeeding and EBF at critical time periods relating to personal and environmental factors are presented in Fig. 1.

### Early initiation of breastfeeding

#### Facilitators of early breastfeeding initiation

All mothers knew about the concept of early initiation of breastfeeding. This is consistent with the fact that PHMs mentioned that health education covering breastfeeding is done at multiple opportunities that during routine antenatal care. According to them, mothers are introduced to the topic of breastfeeding in their second antenatal session in the second trimester of pregnancy and are provided with more detailed information in the third trimester. In the last antenatal domiciliary visit PHMs remind mothers about early initiation and exclusive breastfeeding, and emphasize the importance of adherence.

“We ask mothers to demand the opportunity for early initiation; to hand over the baby and let the child start feeding early as possible, even if the staff in the labor room are very busy.” (a PHM).

The Sri Lankan early initiation of breastfeeding policy has been well implemented in the local hospitals and according to PHMs, it has been implemented with care. They also mentioned that health staff in the hospital labor rooms and operation theaters were dedicated to facilitate early initiation of breastfeeding.

#### Barriers for early breastfeeding initiation

The only reported reason for not practicing early initiation of breastfeeding were medical complications of mother and child. Maternal anaphylaxis and severe postpartum hemorrhage resulting in admission of the mother directly to the intensive care unit, premature delivery (at or before 28 weeks of gestation) and in situations where the child had serious birth complications such as asphyxia, were the reported medical complications in this community. In spite of not being able to follow early initiation of breastfeeding due to these conditions, the maternity ward staff with the help of the lactation management center, ensured that breastfeeding was established before discharging the mother from the hospital.

“I didn’t see my child immediately after birth. As soon as the child was delivered the nurses ran taking him to the PBU [Premature Baby Unit]. I saw my child only after two days. I told them that I want to see my child. They took me to the PBU but I couldn’t see him he was surrounded by tubes... They have given formula milk to the child but I was not informed. Actually, I cannot remember things very well. I was also ill. After two days, I was allowed to express milk and feed the child. I expressed in the ward and send the milk to the PBU. On the fourth day, the baby was given to me and was attached to the breast. While in the ward I listened to the education sessions that were done for mothers on breastfeeding daily by the nurses. Although initially formula milk was given, subsequently I gave only breastmilk.” (mother 03 who underwent emergency cesarean section for a child who was suspected of meconium aspiration).

### Exclusive breastfeeding

#### The time periods of interruption of EBF

Figure 1 shows that time periods when EBF was interrupted in the study sample clustered around three periods. Firstly, within the first 2–3 days, second within the second or third week of delivery, and ultimately around four to five months after birth. The barriers were different at these different time periods and exceeded the driving forces.

### Barriers for EBF around the first 2–3 days of life

We identified maternal, child related and environment related factors that acted as barriers for EBF continuation (Fig. 1).

#### Lack of maternal self-efficacy

Some mothers, who had delivered their first child had many doubts about how to position and attach the child to the breast.

“I didn’t understand how to position the child. The baby did not seem to suckle well. It is only after 2-3 weeks that I got used to hold and attach the baby properly to the breast. After then only that I was satisfied.” (mother 01).

#### Cesarean section

The PHMs reported that EBF was interrupted during the first few days among mothers who underwent cesarean section. One of the PHMs mentioned that they were taught so in their training and that they needed to keep the babies well hydrated. Mothers complained of pain interfering with breastfeeding positioning.

“Most mothers who encounter problems in breastfeeding are mothers who undergo section. We try to promote mothers to go for a normal vaginal delivery as much as possible at the antenatal health education sessions.” (a PHM).

“On the second day I haven’t had enough milk. I could not attach the baby properly to breast. The midwives asked me to hold the child under the arm stating that it will help in attachment. But I could not hold like that as the cesarean section wound was painful and the baby was heavy. I could not lay down on one side either due to pain. I had no milk to express and feed.” (mother 16).

However, we found that even among mothers who had a normal delivery, pain had been a barrier to proper breastfeeding.

“I had a normal vaginal delivery. So, I could not sit. Therefore, I fed the baby in lying down position for the first three weeks.” (mother 01).

#### Social pressure to practice EBF

Some mothers who were well aware of the value of EBF had been anxious about not being able to do so given the strong recommendation coming from the healthcare workers.

“I know that it’s best to give only breast milk. But I was worried that I would not be able to do so.” (mother 16).

#### Suboptimal maternity ward environment

Some ward environments were uncomfortable to mothers. The wards were hot due to the prevailing high temperate in Sri Lanka. The beds in the post-operative wards were high and it was difficult for the mothers to move from bed to chair to feed the child. This challenge was even more pronounced as accompanying person(s) were not allowed even during the first night. The ward staff on duty had to see to every mother’s problems.

“It was a very hot climate and in the ward it was very uncomfortable. I was counting my fingers to leave the hospital. That first night after delivery, almost every newborn had fever. Although fans were there it was unbearably warm. The beds were high. I could not get on the bed alone due to pain. Could not ask for help from the health staff all the time. I was awake all night and was carrying the child. It was very stressful.” (mother 16).

Although most of the mothers and public health staff mentioned that hospital health personnel and the nurses from the lactation management center helped them in establishing breastfeeding, some mothers faced negative comments from them which further reduced their confidence.

“Sometimes they speak without thinking how it would hurt us. I wanted to burp the child and asked for help. The nurse told me that I did not feed my child properly and therefore no point in trying to burp.” (mother 16).

#### Newborn’s medical conditions

Prematurity seemed to be a main reason for introducing infant formula. In some occasions few formula feeds were given initially, but discontinued and practiced EBF after the prematurity-related problems were resolved.

“One of the mothers in my field (allocated area for a PHM) had a premature infant. Initially the hospital asked not to let the baby suckle. So, formula milk was started. But later, the mother started breastfeeding and now she is giving both. I asked the mother to gradually reduce formula feeds and continue breastmilk. I have only this mother infant dyad in my field with disrupted EBF.” (a PHM).

Sometimes problems occurred after coming home from the hospital and the mother was uncertain of the actual reason for the problem.

“I was discharged and at home but on the 3^rd^ day the child became yellow. Even the eyes! I got admitted to the hospital. The health staff said that baby was not fed enough and they gave formula milk. But I had enough milk. though the baby kept sleeping and didn’t suckle for long.” (mother 11).

During the focus group discussions, each PHM described occasional instances they had experienced when infant formula feeds were given in the first 2–3 days on newborns that developed jaundice, fever and multiple deliveries.

“In my field, there were three babies who developed jaundice while in hospital. They were started on formula feeds.” (a PHM).

“One of the mother in my field had a triplet and one of them was not suckling. They consulted a specialist and the child was put on formula feeds.” (PHM).

#### PHMs time constraints for early postpartum home visits

The PHMs do home visits as soon as the mother and newborn are discharge from the hospital to provide domiciliary care. They spent a lot of time troubleshooting and solving early breastfeeding problems. Although PHMs were fully devoted to providing such care, several barriers were identified for the optimal provision of early breastfeeding services.

“For a mother with breastfeeding issues, it takes around 2-3 hours to counsel her, lift her mood and solve the problem. We have to do this even if it is a weekend and a mother needs help. If the mother is having a problem in breastfeeding, we would not be able to fall asleep without solving it. These mothers are helpless after coming home. When we visit them, not only the mother, even the family members are relieved and happy. Regardless of their ethnicity all of them are equally expecting and admiring our service.” (a PHM).

PHMs reported time constraints due to the many other obligations that they have beyond home based breastfeeding support, and sometimes the long distances between the mothers’ homes.

“When we have to cover up vacant areas [an area that does not have a PHM which is covered by a PHM from an adjacent area] and when they are at a distance, sometimes the mothers needing help will be far away from one another. It is very difficult to offer the recommended services when we have cover up duties and I had coverup an additional area since I was appointed. That is for the past whole five years.” (a PHM).

### Facilitators and barriers for EBF at 2–3 weeks postpartum

#### Sub optimal practices of health staff before mother’s discharge

Sometimes mothers with serious medical conditions were trained on cup feeding and at the discharge breastfeeding were not established. This created challenges for the PHMs who had to counsel women on breastfeeding difficulties after hospital discharge.

“I had three newborns in my field who were given formula feeds due to maternal problems. After the mother is stable, they were asked to express the breast milk and feed the child using the cup. When there are maternal complications, they come home after several days. One such mother, I met only on day eight after the delivery. When I visited her at home, she was trying to express milk and was crying! She was unable to attach to child to the breast and was trying to express breast milk all the time. It is exhausting and she had no rest. It is with great difficulty and through mother’s devotion that I was able to establish suckling. One of these mothers actually commenced formula feeds as she could not express enough. They easily go for other options. Therefore, its better if the hospital staff can re-establish child’s attachment to the breast before discharging the mother.” (a PHM).

#### Influence of family members

Negative comments of extended family members affected the mothers’ confidence on breastfeeding leading to introduction of infant formula feeds or feeding water to the child. Comments about “child is not getting enough milk” were mostly made by the mothers in law. Mothers as well as PHMs shared this experience.

“The mother in laws try to force mother to breastfeed the baby too often. They keep complaining that the mother does not have enough milk throughout the day. One of them said that the child should be breastfed as often times a leaf falls from a “tamarind” tree. Ultimately this mother could not bear the situation and went to her own mothers place.” (a PHM).

“When the newborn have hiccups, all the people at home including grandparents get anxious and ask to feed the baby all the time. If a mother is intended to do that she will not have time even to have a bath.” (mother 16).

By contrast, it was also mentioned that the social norms have been changing towards EBF during recent times. According to the mothers and the PHMs, the female adults in the families seemed to accept that breastfeeding practices have now changed for the benefit of the children. They seemed to believe the current knowledge and have faith on PHMs advice.

“No one asked me to give other substitutes at my home. At times of my mother’s delivery, the practice has been to give a glucose solution for the child first. But she knows that now only breastmilk is given. She also asks to get the opinion of the PHM before doing anything new.” (mother 05).

The husbands’ support on EBF was also highlighted during the discussions.

“Since the fathers participate in the antenatal sessions, they know very well about the support that should be given to a mother. Therefore, the husbands support breastfeeding and counteract negative family forces against it. It is a huge benefit.” (a PHM).

#### Past experience, benefits and misconceptions of mothers

Mothers past feeding experience influenced EBF practices. Some mothers talked proudly of their past experiences and how these would help them to continue with EBF for their new child even in the face of some challenges.

“When you hold the child like this [the mother shows the exact positioning] it’s very easy to feed. My sons, they very well-kept suckling until they detach on their own. But this new one [daughter] is not that interested. I sat and fed my sons. But as this one was problematic I tried all positions and somehow kept on feeding.” (mother 05).

Some mothers had strong breastfeeding misconceptions which were properly addressed by the PHMs and specialists with positive messaging.

“Sometimes, even though there is enough breast milk, mothers want to give formula milk to their children. I had one such mother. She was a graduate. She has fed her first child with formula milk due to a medical problem. Now this particular child is doing well and has received national level awards. So, the mother thinks that if she gives formula to the second child, she will be intelligent too. I kept on advising her and forcing her not to do so. Referred her to the MOH. She also consulted a specialist (neonatologist). Listening to them both made her change her mind and adhere to EBF.” (a PHM).

#### Maternal norms and medical practitioners’ prescription of formula feeds

According to the PHMs, some medical practitioners seemed to prescribe formula unnecessarily to mothers. A reason for this may be that in this culture, a “chubby baby” is perceived as a healthy baby. When the babies do not look chubby, they tend to think that its due to inadequate breast milk. This may have led mothers to seek help of medical practitioners and demand to prescribe formula milk.

“The main reason to start formula milk is that when the mothers go and ask for it from the doctors, they just prescribe it. Of course, mothers prefer to listen to what doctors say rather than what we keep saying. Mother is happy as her intention was served by the doctor.” (a PHM).

#### Early introduction of water

Mothers tended to give water during the neonatal period due to several reasons including hot weather, hiccups, and feeling that the child is dehydrated.

“At that time, my baby wanted to get fed every 15-20 minutes. I thought it was due to the dry environment. It was a very hot climate at that time with the drought. .. There was no rain for two years. So, my mother also asked me to give some water to the baby. I only fed two teaspoons at a time.” (mother 15).

“At that time, the child was not getting enough milk and was yellowish and the skin looked dry. Although I didn’t give water at that time, I feel that its better if I had the opportunity to give a little water in a clean manner to get rid of dryness of the child.” (mother 16).

“One child had got frequent hiccups and when mother has consulted a general practitioner he has asked to give a little amount of water when the child is having hiccups.” (a PHM).

### Facilitators and barriers for EBF during 4–5 months postpartum

#### Employed mothers

Returning to work was discussed as the main factor for cessation of breastfeeding before six months after birth.

#### Returning to work

According to the PHMs, most mothers working in the government sector would take the government approved maternity leave of fully paid first four months and half paid next 84 days so that the mother could return to work after introducing complementary foods to the child at six months of age. By contrast, mothers working in other sectors had to report to duty by four months after birth. While some mothers needed to return to work to keep their jobs those in the lower socio-economic groups had to do so to earn money for daily expenses and pay loans.

#### Lack of support after returning to work

Practicing EBF seemed to be difficult when returning to work due to long working hours, long distance between the work place and home, type of job and lack of facilities at the work place to express breastmilk or feed the child.

“One of my employed mothers had started formula milk. She is a bank officer and had to work until late. Another one was a cashier and could only leave her seat to have lunch. It was a small place and she didn’t have a place to express breastmilk during working hours.” (a PHM).

“I like to continue only breast milk. As I don’t get official maternity leave, I have submitted a medical leave these days. Ideally, I have to report for duty after four months. First, I thought of coming home at the lunch interval and feeding the child. But the distance is too far.” (mother 02).

#### Lack of self-efficacy expressing breastmilk

Despite being a main strategy in promoting breastfeeding, feeding the child with expressed breast milk did not seem to be common in the community. Mothers didn’t have confidence to be successful with it.

“Some of the working mothers told me about expressing breastmilk. I was supposed to get advice and learn it from the PHM. But I don’t think I will be able to express a lot in the morning and when the child grows she needs more. So, I decided to start complementary foods.” (mother 02).

#### Introducing complementary foods before six months after delivery

Few mothers introduced water, fruits and some started recommended complementary foods on their own. Some of these actions were due to social influences. The following quotes are from mothers who introduced foods at around four months.

“Although the PHMs ask us to feed until six months there are mothers in the village who gives rice at four months in the same way that they eat. Even though we give meals according to the recommended method our babies are not chubby.” (mother 15).

“I felt that the baby loves bananas. So, I gave some at four months. No one told me to give. I thought the baby will like it.” (mother 08).

#### Misconceptions about on demand breastfeeding among health workers and mothers

Breastfeeding on-demand was one aspect that was found to be challenging. Although the mothers were able to identify their children’s signals of hunger after a few months, they faced many problems learning when to breastfeed during the neonatal period. Health workers including medical officers and practitioners both in the institution and the field advised to feed the child according to a set schedule. Almost every mother mentioned that a child should be fed every two hours. Some mothers thought that feeding on-demand makes them feel exhausted and some expressed that unless the child is awake and fed every two hours, the child will sleep and will not get adequate milk.

“I was asked to feed the child two hourly. But the child does not wait that much. He cries within one hour of the previous feed.” (mother 11).

“My doctor asked me to feed the child every two hours for around half an hour duration.” (mother 10).

## Discussion

Our study documents that even though Sri Lanka is considered as a model to the world for breastfeeding protection, promotion and support, there are still many opportunities to improve the quality of breastfeeding care. This qualitative study is indeed innovative because it identified differences in barriers and facilitators for EBF across time, taking into account the perspectives of PHMs and mothers themselves. Our findings suggest that most of the barriers for EBF can be anticipated and prevented with proper planning and anticipatory guidance. Overall, we specifically found that there is a need to strengthen postnatal ward support during the first 2–3 days, followed by family and public health systems support during the first few weeks after birth. Enhancing maternity benefits and support for breastfeeding for working mothers once they prepare to and return to work will be beneficial. These multi-level factors needed to improve EBF duration need to be addressed through well-coordinated multisectoral programs based on the social-ecological model and healthcare systems frameworks [25].

Early initiation of breastfeeding was frequently followed perhaps as a result of the proper implementation of this BFHI step in the maternity hospitals where the mothers in the study received maternity care [26]. Anticipatory breastfeeding guidance during the antenatal period through the public health system seems to be effective at promoting early initiation of breastfeeding and EBF in the target community. This finding contrasts with some evidence from high income countries suggesting that antenatal breastfeeding education is ineffective [27].

We identified that early initiation of breastfeeding is sometimes not followed due to maternal and neonatal medical problems. Specifically, we found that preventable maternal near misses and newborn emergencies need to be addressed effectively to optimize breastfeeding practices. Our findings also suggest that having safe effective procedures in place for early initiation of breastfeeding when there are delivery complications should be given more priority.

Consistent with previous work [28–30] this study documents that many of the problems related to EBF arise in the maternity facility and tend to remain if the relevant support is not given by the time the mother is discharged. We found that despite the fact that mothers reported benefitting from the routine lactation management support in the hospital, and the care given by the ward staff, support needs to be tailored to the individual needs of mothers experiencing difficulties during early days after birth. Although provision of tailored counseling would be difficult in overcrowded hospitals in, it is key to develop triage protocols to offer to those women who need it the most.

Our study supports the association of cesarean section with interruption of exclusive breastfeeding [30]. Hence policies and practices on deciding the mode of delivery should be carefully re-examined especially in the context of the very high cesarean section delivery rates (39%) observed in Sri Lanka [31]. Although it is encouraging that our study shows that PHMs try to encourage normal vaginal delivery, their efforts have been insufficient to reduce the number of unnecessary C-sections, indicating that reducing cesarean section rates will require a whole health care systems approach.

Violations of the WHO Code for Promotion of Breastmilk Substitutes have been previously reported in urban communities in Sri Lanka [32]. Our findings indicate that medical officers should be better trained and postive attitudes towards promoting EBF need to be emphasized more. The enforcement of the WHO Code indeed needs to be substantially strengthened in Sri Lanka [33].

The involvement and support from husbands since the antenatal sessions is likely to benefit the protection, promotion and support of EBF in Sri Lanka. An interesting finding from our study was the change in negative social norms on EBF among older adults. Yet, influence of family members [34] and social norms such as “a baby needs water in addition to breastmilk” were consistent with findings previously reported elsewhere [35].

We observed that the concept of on-demand breastfeeding was not well grasped by the mothers as well as the healthcare workers. The messages addressing on-demand breastfeeding should be refined and delivered through sound breastfeeding education and counselling in Sri Lanka.

While the prevailing maternity leave policies for government workers are reasonable, Sri Lanka should think of enhancing maternity protection initiatives to all working mothers as it is being done in some high-income countries [36]. Our findings suggest that supporting women to improve their self-efficacy in expressing breast milk should be given priority, which is consistent with existing recommendations [37].

Although the PHMs state that they are satisfied with their training, our findings suggest the central level maternal and child health authorities establish a system of continuous professional development and training specifically on counselling skills [38]. Although educating health staff per se has not been found to improve breastfeeding in high income settings [39], in Sri Lanka this approach is likely to work given the strong government commitment to protect, promote, and support breastfeeding through high quality multisectoral policies and actions [38].

Mothers support groups for breastfeeding is the only BFHI step that has not been strategically implemented in Sri Lanka. However, this will be possible as some of the mothers who tackled early breastfeeding problems ended up having adequate self-efficacy and value expectations (expectation for success and subjective task value) [40] on EBF. Thus, moving forward we recommend establishing well structured breastfeeding mother support groups to improve breastfeeding outcomes in Sri Lanka.

The PHMs faced strong logistical barriers for offering quality breastfeeding services. Consistent with other studies, these barriers included lack of time due to a heavy work load. This was expected given that the work load of PHMs consists of antenatal, postnatal, infant and child care and many other health care aspects. It is important to better estimate the real work loads of PHMs in Sri Lanka to better estimate the case load that they can handle to offer quality breastfeeding counselling services [41]. In addition, proper PHM recruitment and retention procedures are needed to reduce the excessive work load the PHMs have often driven by the fact that they need to cover the demand in areas that are vacant (i.e., without a PHM).

This study was conducted in Anuradhapura district which is predominantly rural. Hence our results cannot be generalized to higher income urban, estate or shanty population in Sri Lanka. However, Sri Lanka as well as many other lower income countries may have similar rural populations for which our findings could be generalized [42]. Therefore, this study provides evidence to better inform effective health system responses to protect, promote and support EBF among rural populations in lower income countries. When interpreting our findings, it is important to note that we only interviewed only mothers who were not successful with early initiation of breastfeeding or exclusive breastfeeding. Further qualitative studies that include women who were successful with early initiation of breastfeeding and EBF may provide additional insights.

## Conclusions

Interruption of EBF clusters around the first 2–3 days, first few weeks and around 4–5 months after birth with specific barriers corresponding to the different time periods. The reasons for interruption of early initiation of breastfeeding were mostly related to serious medical conditions among mothers and newborns. Exclusive breastfeeding interruption may be prevented by facilitating and enabling the breastfeeding environments rooted in the social-ecological model. Sri Lanka should focus on reducing the excessive rates of cesarean section, promoting better breastfeeding support in maternity wards, promoting on-demand breastfeeding, offering better support for working mothers to continue EBF up to six months, and strengthening the ability of PHMs to deliver high quality breastfeeding services [43].

## Supplementary Information

**Additional file 1.** Appendix 1

**Additional file 2.** Appendix 2

**Additional file 3.** Appendix 3

**Additional file 4.** Appendix 4

## Data Availability

Data could be made available on request by the corresponding author.

## References

[1] Raisler J, Alexander C, O’Campo P (1999). Breast-feeding and infant illness: a dose-response relationship?. Am J Public Health.

[2] World Health Organization. *WHO Exclusive breastfeeding: Infant young child Feeding fact sheet*. World Health Organization. 2016. Available from: https://www.who.int/en/news-room/fact-sheets/detail/infant-and-young-child-feeding. Accessed 31 December 2020.

[3] Mullany LC, Katz J, Li YM, Khatry SK, Leclerq SC, Darmstadt GL (2009). Breast-feeding patterns, time to initiation, and mortality risk among newborns in southern Nepal. J Nutr.

[4] Anderson JW, Johnstone BM, Remley DT (1999). Breast-feeding and cognitive development: a meta-analysis. Am J Clin Nutr.

[5] Sankar MJ, Sinha B, Chowdhury R, Bhandari N, Taneja S, Martines J, Bahl R (2015). Optimal breastfeeding practices and infant and child mortality: a systematic review and meta-analysis. Acta Paediatr.

[6] Horta BL, Victora CG (2013). Short-term effects of breastfeeding: a systematic review of the benefits of breastfeeding on diarrhoea and pneumonia mortality.

[7] Bowatte G, Tham R, Dai X (2015). Breastfeeding and childhood acute otitis media : a systematic review and meta-analysis. Acta Paediatr.

[8] Lodge CJ, Tan DJ, Lau MXZ, Dai X, Tham R, Lowe AJ, Bowatte G, Allen KJ, Dharmage SC (2015). Breastfeeding and asthma and allergies: a systematic review and meta-analysis. Acta Paediatr.

[9] Horta BL, Mola CL, Victora CG (2015). Breastfeeding and intelligence: a systematic review and meta-analysis. Acta Paediatr.

[10] Horta BL, Mola CL, Victora CG (2015). Long-term consequences of breastfeeding on cholesterol, obesity, systolic blood pressure and type 2 diabetes: a systematic review and meta-analysis. Acta Paediatr.

[11] Weimer JP. *The economic benefits of breastfeeding: A review and analysis (no.13).* US Department of Agriculture, Economic Research Service; 1999.

[12] United Nations International Children’s Emergency Fund. *Breastfeeding a matter of human rights, say UN experts, urging action on formula milk. 2016*. Available from: https://www.ohchr.org/EN/NewsEvents/Pages/DisplayNews.aspx? NewsID=20904&LangID=E. .

[13] The World Breastfeeding Trends Initiative (WBTi)*. Press Briefing: The World Breastfeeding Trends Initiative (WBTi), Congratulates Sri Lanka on achieving first “Green” nation status supporting breastfeeding women. 2020*. p. 4–6. Available from: https://www.worldbreastfeedingtrends.org/uploads/resources/document/wbti-press-release-9-jan-2020.pdf. Accessed 31 December 2020.

[14] World Bank. New country classification according to income 2020–2021. 2020. Available from: https://blogs.worldbank.org/opendata/new-world-bank-country-classifications-income-level-2020-2021. .

[15] Ministry of Health Sri Lanka. *National Maternal and Child Health Policy of Sri Lanka*. Colombo; 2012.

[16] Food and Nutrition Policy Planning Division, Ministry of Planning Implementation G of SL. *Sri Lanka code for the promotion of breast feeding and marketing breast milk substitutes and related products.* Colombo; 1983.

[17] Wickramasinghe SC (2012). Lactation management centres: a step forward in successful breast feeding. Sri Lanka J Child Health.

[18] Department of Census and Statistics, Ministry of National Policies and Economic Affairs. *Sri Lanka Demographic and Health Survey* 2016*.* Colombo; 2017.

[19] Department of Census and Statistics, Ministry of Policy Planning Economic Affairs, Child Youth and Cultural Affairs Sri Lanka. *Census on population and housing 2012*. Colombo; 2015. Available from: http://www.statistics.gov.lk/PopHouSat/Mid Year Population/midyearsex&district.pdf. Accessed 31 December 2020. Accessed 31 December 2020.

[20] Ministry of Healthcare and Nutrition Sri Lanka. *Annual Report of the Family Health Bureau*. Colombo; 2018.

[21] Mack N, Woodsong C, Macqueen KM, Guest G, Namey E. Indepth interviews. In: *Qual. Research Methods: A Data Collector Field Guide.* North Carolina, USA: Family Health International; 2011.

[22] Mack N, Woodsong C, MacQueen KM., Guest G, Namey E. *Qualitative Research Methods: A Data Collector’s Field Guide.* 2011*.* North Carolina, USA: Family Health International; 2011.

[23] Lewin K. *Field theory in social science: selected theoretical papers.* Cartwright D, editor. New York; 1951.

[24] Bandura A (1971). Social learning theory.

[25] Pérez-Escamilla R, Curry L, Minhas D, Taylor L, Bradley E (2012). Scaling up of breastfeeding promotion programs in low- and middle-income countries: “breastfeeding gear model”. Adv Nutr.

[26] Pérez-Escamilla R, Martinez JL, Segura-Pérez S (2016). Impact of the baby-friendly hospital initiative on breastfeeding and child health outcomes: a systematic review. Matern Child Nutr.

[27] Lumbiganon P, Martis R, Laopaiboon M, Festin MR, Ho JJ, Hakimi M (2016). Antenatal breastfeeding education for increasing breastfeeding duration. Cochrane Database Syst Rev.

[28] Hobbs AJ, Mannion CA, McDonald SW, Brockway M, Tough SC (2016). The impact of caesarean section on breastfeeding initiation, duration and difficulties in the first four months postpartum. BMC Pregnancy Childbirth.

[29] Prior E, Santhakumaran S, Gale C, Philipps LH, Modi N, Hyde MJ (2012). Breastfeeding after cesarean delivery: a systematic review and meta-analysis of world literature. Am J Clin Nutr.

[30] Perez-Escamilla R, Maulen-Radovan I, Dewey KG (1996). The association between cesarean delivery and breast-feeding outcomes among Mexican women. Am J Public Health.

[31] Family Health Bureau of the Ministry of Healthcare and Nutrition Sri Lanka. *Statistics.* 2018. Available from: https://fhb.health.gov.lk/index.php/en/statistics. Accessed 31 December 2020.

[32] Agampodi SB, Agampodi TC, Piyaseeli UKD (2007). Breastfeeding practices in a public health field practice area in Sri Lanka: a survival analysis. Int Breastfeed J.

[33] Dennis CL, Brown HK, Chung-Lee L, Abbass-Dick J, Shorey S, Marini F, Brennenstuhl S (2019). Prevalence and predictors of exclusive breastfeeding among immigrant and Canadian-born Chinese women. Matern Child Nutr..

[34] Houghtaling B, Byker Shanks C, Ahmed S, Rink E (2018). Grandmother and health care professional breastfeeding perspectives provide opportunities for health promotion in an American Indian community. Soc Sci Med.

[35] Swigart TM, Bonvecchio A, Théodore FL, Zamudio-Haas S, Villanueva-Borbolla MA, Thrasher JF. Breastfeeding practices, beliefs, and social norms in low-resource communities in Mexico: insights for how to improve future promotion strategies**.** PLoS One 2017; 12(7): e0180185. https://doi.org/10.1371/journal.pone.0180185. 10.1371/journal.pone.0180185 PMC5495390 28671954

[36] Employment and Social Development Canada. *Labour Code Canada; Maternity-related reassignment and leave, maternity leave and parental leave. 2017*. Available from: https://www.canada.ca/content/dam/esdc-edsc/documents/services/reports/No.1176-Labour Standard 5-EN.pdf. Accessed 31 December 2020.

[37] Dinour LM, Szaro JM (2017). Employer-based programs to support breastfeeding among working mothers: a systematic review. Breastfeed Med.

[38] Agampodi SB, Agampodi TC (2008). Effect of low cost public health staff training on exclusive breastfeeding. Indian J Pediatr India.

[39] Feltner C, Weber RP, Stuebe A, Grodensky CA, Orr C, Viswanathan M. *Breastfeeding Programs and Policies, Breastfeeding Uptake, and Maternal Health Outcomes in Developed Countries.* Rockville (MD): Agency for Healthcare Research and Quality (US); 2018 Jul. Report No.: 18-EHC014-EF. PMID: 30204377. 30204377

[40] Petersen J, Hyde JS (2014). Gender-related academic and occupational interests and goals. Adv Child Dev Behav.

[41] Kavle JA, Picolo M, Buccini G, Barros I, Dillaway CH, Pérez-Escamilla R**.** Strengthening counseling on barriers to exclusive breastfeeding through use of job aids in Nampula, Mozambique**.** *PLoS One* 2019; 14(12): e0224939. https://doi.org/10.1371/journal.pone.0224939. 10.1371/journal.pone.0224939 PMC6886792 31790430

[42] Smith J, Cattaneo A, Iellamo A, Javanparast S, Atchan M, Gribble K et al. Review of Effective Strategies to Promote Breastfeeding. The Sax Institute, 2018.

[43] Chapman DJ, Morel K, Anderson AK, Damio G, Pérez-Escamilla R (2010). Breastfeeding peer counseling: from efficacy through scale-up. J Hum Lact.

