# Supplementary material for: Barriers for early initiation and exclusive breastfeeding up to six months in predominantly rural Sri Lanka: a need to strengthen policy implementation
Source: Int Breastfeed J. 2021 Apr 8;16:32. doi: 10.1186/s13006-021-00378-0 (PMC8034146; doi:10.1186/s13006-021-00378-0)
Supplement: Supplementary file 1 — Additional file 1. Appendix 1 [file 13006_2021_378_MOESM1_ESM.docx]

Appendix 1: The event calendar for assessment of introduction of substitutes during the first six months of infancy.

Please indicate whether you have given any of following to your baby and indicate the time.

|  | Given | Age of the baby in months at that time |
| --- | --- | --- |
| Water |  |  |
| Rata kalka (an aurvedic medicine) |  |  |
| Rice kanji (watery) |  |  |
| Rice in semisolid form |  |  |
| Soup (thick) |  |  |
| Fruit juice |  |  |
| Coriander water |  |  |
| Formula milk |  |  |
| Other liquid or solid foods  (please specify) |  |  |
