# Supplementary material for: Barriers for early initiation and exclusive breastfeeding up to six months in predominantly rural Sri Lanka: a need to strengthen policy implementation
Source: Int Breastfeed J. 2021 Apr 8;16:32. doi: 10.1186/s13006-021-00378-0 (PMC8034146; doi:10.1186/s13006-021-00378-0)
Supplement: Supplementary file 2 — Additional file 2. Appendix 2 [file 13006_2021_378_MOESM2_ESM.docx]

| Appendix 4: Interviewer guide with PHM on EI and EBF status and barriers in her area | |
| --- | --- |
| Attitudes about knowledge of mothers about early initiation of breast feeding | I’m going to ask some questions about the status of EI and EBF of mothers under your care.  What do you think about the current knowledge of mothers regarding early initiation of breast feeding and solving of problems regarding that? |
|  | How and when would you give that knowledge to mothers? |
| Attitude on practices | Are there infants who missed initiation of breast feeding in first hour in your area? |
|  | What do you think the reason for that? |
| Attitudes about knowledge of mothers about exclusive breast feeding | What do you think about the knowledge regarding exclusive breast feeding of first six months and solving of problems regarding that? |
|  | How and when would you give the knowledge about exclusive breast feeding of first six months to mothers? |
| Attitude on practices | Are there infants with non- exclusive breast feeding in your area? |
|  | What do you think the reason for that? |
|  | What are the other types of food that was given to infants other than breast milk? |
| Challenges faced | Can you explain the problems and challenges that you have to face while providing knowledge? |
|  | What are the measures you have taken to solve problems arise here? |
|  | Can you explain the problems and challenges that you have to face while solve those problems? |
|  | How do you assess whether mothers are following your advices? |
