# Supplementary material for: Barriers for early initiation and exclusive breastfeeding up to six months in predominantly rural Sri Lanka: a need to strengthen policy implementation
Source: Int Breastfeed J. 2021 Apr 8;16:32. doi: 10.1186/s13006-021-00378-0 (PMC8034146; doi:10.1186/s13006-021-00378-0)
Supplement: Supplementary file 3 — Additional file 3. Appendix 3 [file 13006_2021_378_MOESM3_ESM.docx]

| Appendix 2: Interviewer Guide for mothers who could not practice Early Initiation (EI)  of breastfeeding (BF) in first hour of life | |
| --- | --- |
| Information of mother | Age |
| Serial number: | Educational level |
|  | Occupation |
|  | Family income |
| Mothers’ knowledge | I’m going to ask you few questions on BF. Do you think it is important to start breastfeeding in the first hour of life? Why do you think so? |
|  | Who informed you and explained you about this? When did they do that? (service of MOOH and PHMs) |
| Mothers’ practices and influencing factors | Can you remember the day your child was born? Can you please describe it for me? (Information about the delivery, whether normal vaginal delivery or Cesarean section, planned or emergency) |
|  | Can you please describe me how you started breast feeding to your child? What actually happened on that day? (know whether breast feeding started in first hour)  After that what happened? Who helped you? |
| Barriers | Can you please describe me the problems you faced in EI to your child? |
|  | Did you get any help for Initiation of breast feeding in first hour of life to your child? Who helped you? |
| Attitudes | What was the attitude of your family and surrounding about Initiation of breast feeding in first hour of life? |
