# Supplementary material for: Barriers for early initiation and exclusive breastfeeding up to six months in predominantly rural Sri Lanka: a need to strengthen policy implementation
Source: Int Breastfeed J. 2021 Apr 8;16:32. doi: 10.1186/s13006-021-00378-0 (PMC8034146; doi:10.1186/s13006-021-00378-0)
Supplement: Supplementary file 4 — Additional file 4. Appendix 4 [file 13006_2021_378_MOESM4_ESM.docx]

| Appendix 3: Interviewer Guide for mothers who had interruption in Exclusive breast feeding (EBF) before completion of first six months of the infant. | |
| --- | --- |
| Information of mother | Age |
|  | Occupation |
|  | Educational level |
|  | Family income |
| Knowledge | I’m going to ask you few questions on BF. Do you think EBF up to six months is important for the child? Why do you think so? |
|  | Who informed you and explained you about EBF? When did they do that? (service of MOOH and PHMs) |
| Practices | How would you decide the time that your child needs breast feeding? What do you do at that time? |
|  | Are there instances you had to give foods other than breast milk to your child? (water, syrup) |
|  | Why you had to give foods other than breast milk to your child? |
|  | Have you given infant formula milk to your child? (Who suggested giving infant formula milk?) |
|  | What do you think about giving food additional to breast feeding to your child in first 6 months? |
|  | What type of problems did you encounter at home? |
| Attitudes | What is the attitude of your family and surrounding about exclusive EBF upto first 6 months? |
